# Themes and trends in marathon performance research: a comprehensive bibliometric analysis from 2009 to 2023

**DOI:** 10.3389/fphys.2024.1388565

**Published:** 2024-05-10

**Authors:** Liping Yan, Ziyan Chen, Xue Zhang, Qi Han, Jingyi Zhu, Qirong Wang, Zhiguang Zhao

**Affiliations:** ^1^ Institute of Medical Information, Chinese Academy of Medical Sciences, Beijing, China; ^2^ College of Public Health, Zhengzhou University, Zhengzhou, China; ^3^ Sports Nutrition Center, National Institute of Sports Medicine, Beijing, China; ^4^ Key Lab of Sports Nutrition, General Administration of Sport of China, Beijing, China

**Keywords:** marathon, performance, Bibliometrics, athletes, review

## Abstract

**Background:** When marathon runners break the 2-h barrier at the finishing line, it attracts global attention. This study is aimed to conduct a bibliometric analysis of publications in the field of marathon running, analyze relevant research contributors, and visualize the historical trends of marathon performance research over the past 15 years.

**Methods:** On 8 December 2023, we extracted high-quality publication data from the Web of Science Core Collection spanning from 1 January 2009 to 30 November 2023. We conducted bibliometric analysis and research history visualization using the R language packages biblioshiny, VOSviewer, and CiteSpace.

**Results:** A total of 1,057 studies were published by 3,947 authors from 1,566 institutions across 63 countries/regions. USA has the highest publication and citation volume, while, the University of Zurich being the most prolific research institution. Keywords analysis revealed several hotspots in marathon research over the past 3 years: (1) physiology of the elite marathon runners, (2) elite marathon training intensity and pacing strategies, (3) nutritional strategies for elite marathon runners, (4) age and sex differences in marathon performance, (5) recovery of inflammatory response and muscle damage.

**Conclusion:** This study presents the first comprehensive bibliometric analysis of marathon performance research over the past 15 years. It unveils the key contributors to marathon performance research, visually represents the historical developments in the field, and highlights the recent topical frontiers. The findings of this study will guide future research by identifying potential hotspots and frontiers.

## Background

When marathon runners break world records of 2-h barrier, it will likely attract significant global attention (Joyner et al., 2011; Hoogkamer et al., 2017). As reported by the International Association of Athletics Federations (IAAF), Kelvin Kiptum and Tigst Assefa set new world records with completion times of 02:00:35 and 02:11:53, respectively, in 2023 (Competition Performance Rankings, 2024). These exceptional and outstanding achievements in this field directing renewed interest in improving marathon performance.

Previous research established that peak oxygen uptake, anaerobic threshold velocity, and running economy play pivotal roles in endurance performance (Joyner and Coyle, 2008; van der Zwaard et al., 2021) as well as marathon performance (Jones et al., 2021; Haugen et al., 2022). Elite marathon runners are highly skilled and well-conditioned athletes characterized by exceptional running economy and observed with cardiovascular and cardiopulmonary capacity. Historically, the majority of marathon performance investigations were conducted within topics of physiological bio-markers associated with these core metrics, including anthropometry, heart size, hemoglobin content, and capillary density among top marathon runners (Billat et al., 2001; Joyner and Coyle, 2008). Recent research has delved deeper, revealing that 35 years old of age marks the pinnacle of endurance performance for proficient marathon runners (Lepers et al., 2020). Additionally, high altitude training and high-intensity exercise lead to an increase in endomembrane folds and enhanced mitochondrial efficiency in the lower extremity muscles of elite endurance athletes (King, 2021). Furthermore, the discovery of veillonella, a microorganism leveraging lactate metabolism to bolster performance, has provided insights at the microscopic level (Scheiman et al., 2019). Studies exploring the interaction between external environment factors and marathon performance particularly performance over longer distances are increasing. Notably, in August 2023, physicists unveiled that a grouping of seven pacers arranged in a three-swordfish formation could reduce air resistance for the targeted pacer by approximately 60%, thus enhancing performance (Physicists find a way to set a new marathon record, 2023). Advances in footwear technology aimed at enhancing the running economy have also garnered significant attention in recent years (Muniz-Pardos et al., 2021). Investigations into the impact of weather conditions have revealed that elite runners accelerate as humidity and daylight hours increase (Weiss et al., 2022). The factors influencing marathon performance are intricate and multifaceted, underscoring the criticality of staying abreast of the latest information for the scientific training of marathon runners. Researchers must identify prevailing hot research topics and future trends, and given the proliferation of marathon performance research, the adoption of novel approaches to literature analysis has become indispensable.

Bibliometrics, a methodology that qualitatively and quantitatively analyzes publications in a specific field, allows for the identification of influential countries, authors, institutions, journals, and publications (Donthu et al., 2021). Furthermore, it enables the analysis of the knowledge structure within the field and the exploration of cutting-edge research hotspots and trends (Mukherjee et al., 2022). In the realm of kinesiology, bibliometric studies are limited, and no comprehensive analysis of marathon has been conducted.

This study employs bibliometric methods to comprehensively analyze and summarize the most influential countries, institutions, authors, publications, and keywords in marathon performance literature from the past 15 years, identifying the development of hotspots and trends in marathon performance research.

## Materials and methods

### Data source and search strategy

**FIGURE 1:**
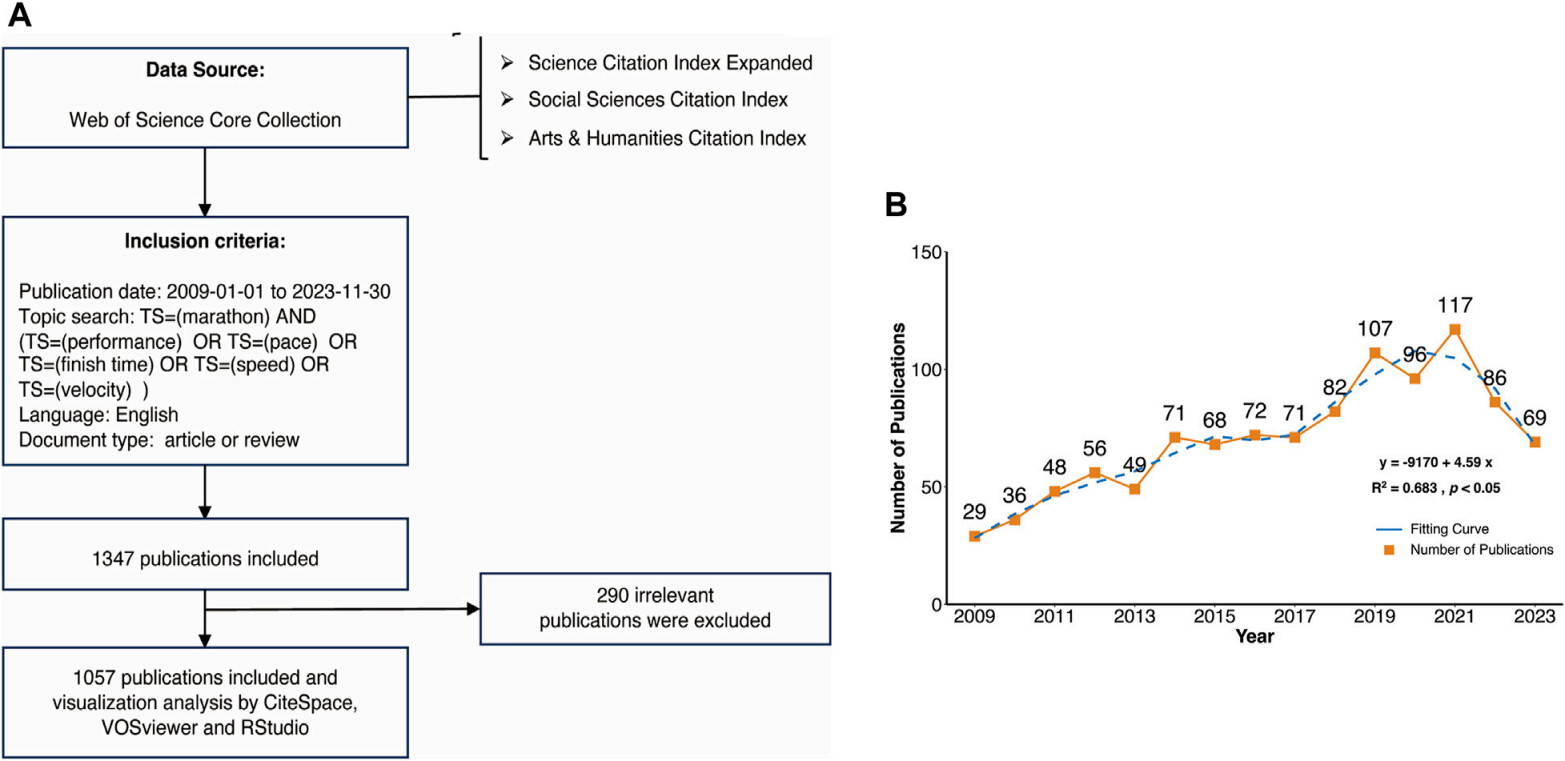
**(A)** The data collection and search strategy. **(B)** Annual publications in marathon performance research during 2009-2023.

In this study, we mainly searched three high-quality citation databases, SCI-Expanded, SSCI, and A&HCI, in the Web of Science Core Collection (WOSCC) database platform, the search time is up to 2023-11-30. The specific search strategy is illustrated in Figure 1A. Data inclusion and exclusion criteria were as follows:

(1) The search interval was from 2009-01-01 to 2023-11-30.
(2) The search strategy was “TS=(marathon) AND (TS=(performance) OR TS=(pace) OR TS=(finish time) OR TS=(speed) OR TS=(velocity))";
(3) The search type was “article” and “review";
(4) The search language was “English".

Based on our search results, we identified 1,347 papers. Upon reviewing the titles and abstracts of these papers, we excluded 290 that were deemed irrelevant to our research. Finally, we obtained a total of 1,057 documents comprising 988 articles and 69 reviews.

### Data analysis and visualization

Data analysis and visualization were conducted using various tools and techniques. The R language package biblioshiny, VOSviewer, and CiteSpace were employed to investigate various facets of the research.

Specifically, the biblioshiny tool served as the primary instrument for the comprehensive summarization and organization of data pertaining to marathon performance research. This encompassed the analysis of annual publications, predominant countries, institutions, and individual authors, along with the top 10 co-cited references. Additionally, biblioshiny facilitated the synthesis of information on the top 30 most frequently occurring keywords and their distribution across the years 2009–2023. It further aided in examining the thematic evolution of keywords in the year 2023. VOSviewer played a pivotal role in visualizing collaboration patterns among countries, institutions, and authors. It also facilitated the depiction of co-occurrence networks pertaining to keywords. Meanwhile, CiteSpace was predominantly utilized for identifying research frontiers through the application of the citation burst function, unveiling influential literature and keywords.

## Results

### Number of publications over the years

The annual literature production reflects the dynamics of research development. Figure 1B shows that the overall marathon performance research has increased steadily at an annual growth rate of 6.39%. The number of publications per year was below 70 until 2014, and then rose sharply after 2018, reaching a peak of 117 in 2021. However, the fitting of the variables ‘publications’ and ‘year’ using the loess function (*R* ^2^ = 0.683, *p* < 0.05) revealed a slight decrease in research output in this field over the last 5 years.

### Countries/regions distribution in marathon performance

The Marathon Performance Study involved researchers from 63 countries/regions. Supplementary Table S1 displays the 10 countries with the most publications, with the United States leading (652 publications, 5,133 citations), followed by Switzerland (423 publications, 3,122 citations) and Spain (335 publications, 1,208 citations). The co-authorship analysis of countries/regions reveals international collaborations.Supplementary Figure S1 uses VOSviewer’s built-in function co-authorship analysis to analyze the collaboration among 28 countries/regions with over 10 publications. Co-authorship analysis can unveil the strength and level of connection among various entities by examining the frequency and patterns of collaboration among countries, institutions, and authors who have jointly authored papers (Donthu et al., 2021). The results reveal a division of the cooperation network into four distinct color clusters, with the red and green clusters being the largest and centered on the United States and Switzerland, respectively. Specifically, the United States primarily collaborates with countries/regions including England and Australia, whereas Switzerland’s main collaborators are countries/regions such as France and Greece.

### Institutions distribution in marathon performance

As depicted in Supplementary Figure S2, a total of 1,566 institutions have engaged in marathon performance issuance, with 36 institutions producing more than 10 articles. Among these, the University of Zurich in Switzerland emerges as the leading contributor, generating 174 articles, followed by the University of West Attica with 30 articles. Co-authorship analysis of 119 institutions, each with a publication frequency exceeding 5, was conducted using VOSviewer. The findings depicted in Supplementary Figure S3 demonstrate that the collaborative network is segmented into eight distinct color clusters. Notably, the largest and most interconnected cluster, represented by purple, is centered on the University of Zurich. This cluster exhibits rich collaborations and maintains closely-knit relationships with several institutions, such as the University of Western Attica and the University of Malaga.

### Authors distribution in marathon performance

The contribution of scholars with a high volume of publications and citation frequency is crucial for the advancement of the field of marathons. Supplementary Table S2 displays the top 10 authors in the field of marathon performance based on publication volume, along with their corresponding H-index and citation frequency. The H-index is a crucial metric for evaluating the academic impact of scholars. Higher values signify a more significant impact, calculated mainly using all of an author’s publications. Over the past 15 years, marathon performance research has been primarily led by 3,947 authors, with Knechtle B (171 articles) emerging as the top scholar in terms of publication count, H-index, and citation frequency. VOSviewer was employed to analyze co-authorship patterns of 89 authors with a frequency greater than 5. Supplementary Figure S4 presents the results, depicting a collaboration network divided into eight clusters represented by different colors, with the largest collaboration cluster in green consisting of 10 authors centered on Knechtle B, Rosemann T. Among these collaborations, Knechtle B has the highest number of partners, totaling 29.

### The top 10 most co-cited references in marathon performance

Co-citation analysis is a scientific mapping technique that posits publications frequently cited together as thematically similar. This method is effective for identifying the most influential publications within a dataset (Cheuvront et al., 2005; Knechtle et al., 2020). Supplementary Table S3 presents the top 10 most co-cited references in the field. The most frequently cited reference, with a total of 67 citations in our dataset, is Lepers R et al., ‘s 2012 publication in the journal of AGE. This study explored that master runners (men aged ≥65 years and women aged ≥45 years) may not yet have reached the limits of marathon performance (Lepers and Cattagni, 2012). A reference by Matthew, comprising 60 citations in our dataset, indicates that marathon running tends to decelerate in warm weather (Ely et al., 2007). The remaining references predominantly examine various aspects of marathon performance, including the impact of factors such as body composition, training techniques, pacing strategies, gender differences, and temperature regulation. Altmetric attention scores reflect research impact, social influence, and dissemination effectiveness (van der Zwaard et al., 2020). Among the top 10 most co-cited references, the American College of Sports Medicine’s studies on exercise and fluid replacement, gender differences in the age of elite marathon runners, and the impact of weather on marathon events demonstrate significant influence and academic attention within both the public and scholarly communities.

### The top 30 most occurrences keywords and their distribution over 2009-2023

**FIGURE 2:**
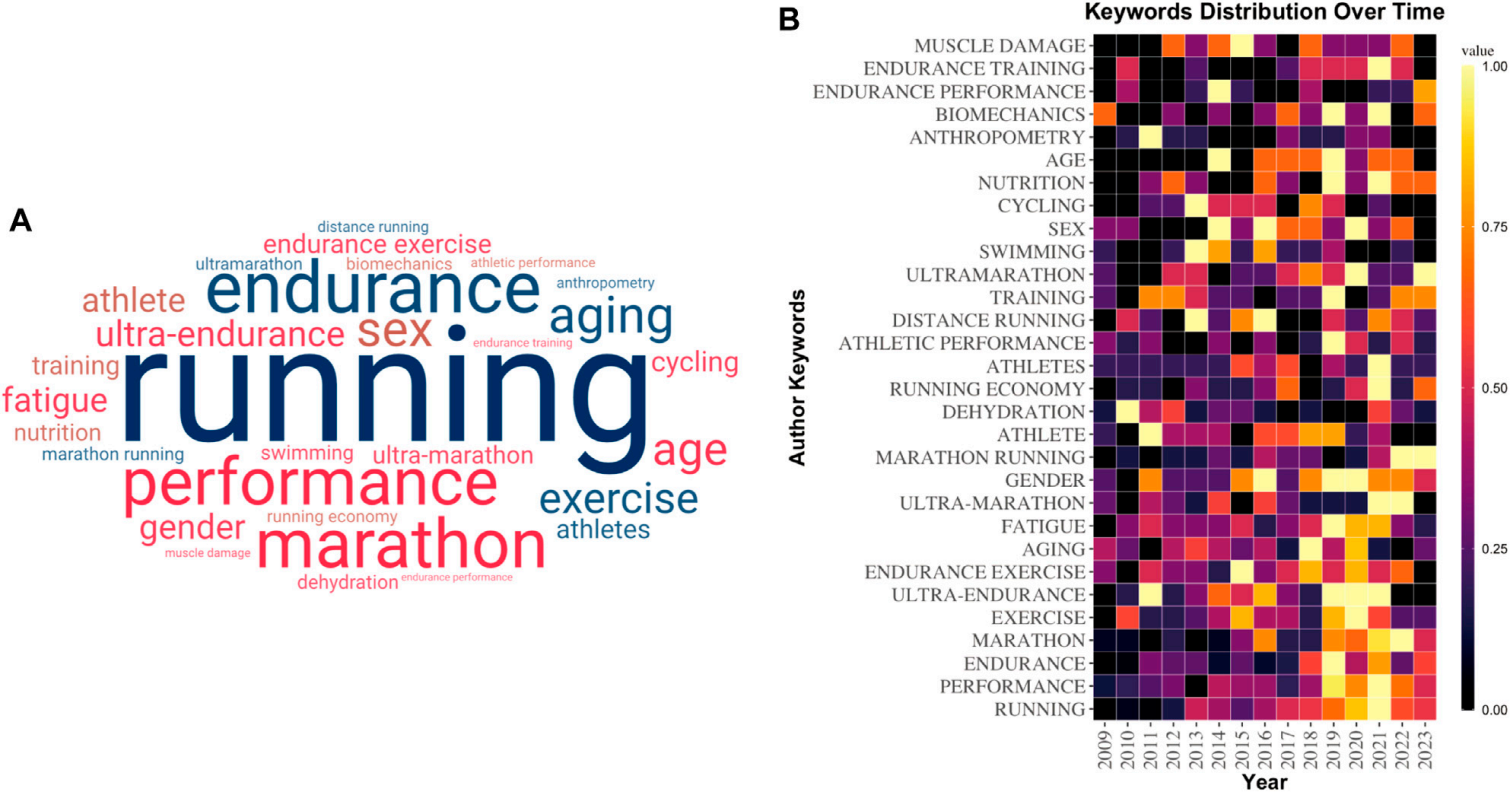
**(A)** The top 30 occurrences keywords word clouds. **(B)** The top 30 most occurrences keywords distribution over 2009-2023.

Keyword frequency serves as an indicator of research hotspots to a certain extent. We utilized the biblioshiny package in R to extract the 30 most frequently occurring author keywords and simultaneously generated the word cloud map using R, depicted in Figure 2A. Among these keywords, “running”, “performance”, “endurance”, and “marathon” emerge as the most prominent, with frequencies of 196, 119, 113, and 98 times, respectively. In Figure 2B, the frequency trends of these 30 author keywords over the last 15 years are depicted by using R package ggplot2. It is evident from the figure that these keywords were less prevalent before 2016. Notably, studies on marathon performance, particularly those related to ultra-marathons, gender, running economy, training, age, and nutrition, have gained increased prominence in the last 3 years, spanning from 2021 to 2023.

### Keywords co-occurrence and thematic evolution in marathon performance

Keywords co-occurrence analysis is primarily utilized to examine the concurrent frequency of two or more keywords in a specified dataset, unveiling the semantic correlations among keywords, and pinpointing the research theme (Donthu et al., 2021). Supplementary Figure S5 illustrates the results of the co-occurrence analysis conducted using VOSviewer on 53 keywords with a frequency greater than 10. The co-occurrence network is segmented into five distinct clusters. The largest cluster, denoted by the red color, predominantly encompasses studies on marathon performance, economy of motion, and the risks associated with sports injuries, such as fatigue, inflammation, muscle injury, and dehydration. In contrast, the green cluster primarily focuses on analyzing the effects of marathon running on echocardiography, heart rate, and other relevant factors. The blue cluster pertains to research investigating differences in athletic performance by gender and age in marathon runners. The yellow cluster explores the relationship between marathon performance and anthropometry as well as body composition. Lastly, the purple cluster delves into studies on marathon biomechanics and stride frequency.

The thematic evolution analysis of 53 keywords with a frequency greater than 10 is depicted in Supplementary Figure S6. This analysis was conducted using the Walktrap Clustering Algorithm in biblioshiny. The Weight index was utilized as an inclusion index, weighted by word occurrence. This analysis provides insights into the temporal trends of research interests within the field. Each node in the Sankey plot corresponds to the keyword with the highest frequency of occurrence, and its size is directly proportional to the number of keywords associated with the topic. The flow of nodes signifies the direction of the evolution of the topic complex, visually depicting the overall flow and transition of themes across categories over time (Schmidt, 2008). The Sankey diagram depicts a stable and compact shift in keyword hotspots, with many current studies deriving from previous periods. For instance, the research on marathon runners’ heart rates was a prominent research focus from 2009 to 2015, persisting until 2016 to 2020. Additionally, the study of pacing strategies and inflammatory responses in marathon runners emerged as a research hotspot from 2009 to 2015 and regained attention as a hotspot from 2021 to 2023. Notably, research conducted in the past 3 years has focused on age-related performance decline, gender differences, pacing strategies, inflammation, and recovery, indicating their status as trending topics.

### References bursts and keywords bursts in marathon performance

The identification of reference bursts and keyword bursts primarily relies on CiteSpace’s built-in burst detection algorithm, employing a gamma value and a minimum duration of 1.0. Reference bursts can facilitate the identification of key literature and keywords in the field of marathon running. The red line segments in Supplementary Figure S7 indicate the time periods of literature/keyword bursts, while strength measures the intensity of the bursts. The figure displays the top 15 references with the highest citation bursts, among which the 2012 article on male (≥65 years) and female (≥45 years) master runners did not reach their limits of performance has the strongest burst (Lepers and Cattagni, 2012). Most of the references have reached their peak citation rates by 2018, except for four recent studies that remain influential: Hoogkamer et al. (2018) studied the effects of different running shoes on energy expenditure (Hoogkamer et al., 2018), Knechtle and Nikolaidis (2018) performed a review of ultramarathon physiology and pathophysiology (Knechtle and Nikolaidis, 2018), Scheer (2019) observed the exponential growth of ultra-marathon participation and the increasing involvement of women (Scheer, 2019), and Vitti et al. (2020) on the rising marathon participation in New York City over the last 50 years, with more significant trends among women and the best performances by Ethiopians and Kenyans (Vitti et al., 2020).

Supplementary Figure S8 presents the top 20 keywords associated with the citation outbreak. Among these keywords, “running performance” stands out as the keyword with the highest outbreak intensity in marathon performance field, aligning with the focus of our research. Additionally, keywords such as “sex differences”, “intensity”, “nutrition”, “running economy”, “health”, “physiology”, and “impact” continue to receive significant attention as hot topics within the field in 2021-2023.

## Discussion

### General distribution

This study conducts a bibliometric analysis of research papers on marathon performance spanning the past 15 years. The analysis reveals a consistent growth trend in the annual number of publications on marathon performance. Notably, there was a significant surge in publications from 2009 to 2014, although a decline was observed in the last 2 years. A total of 3,947 authors affiliated with 1,566 institutions across 63 countries/regions contributed to marathon performance research articles, underscoring widespread scholarly interest. The collaboration network, spanning countries/regions, institutions, authors, and scholars, reflects extensive academic exchanges and cooperation, breaking geographical constraints. The United States emerges as the leading country in both publications and citations. The University of Zurich and Knechtle B from the University of Zurich stand out as the most productive institutions and scholars, respectively. Knechtle B is further recognized as the most influential scholar and a frequent collaborator in the field. His 2018 review on ultramarathon physiology and pathophysiology is a top 15 reference with the strongest citation bursts, garnering sustained attention (Knechtle and Nikolaidis, 2018).

### Research hotspots and trends

Based on keyword occurrences, time distribution, co-occurrence, thematic evolution, citation bursts analysis, and the results of a comprehensive analysis of research hotspots in the past 3 years, we have organized the findings into the following thematic chapters.

### Physiology of the elite marathon runners

Maximum oxygen uptake (peakVO_2_), anaerobic threshold speed, and running economy are three crucial factors that impact marathon performance (Joyner and Coyle, 2008; Haugen et al., 2022). In 2017, a study demonstrate a peakVO_2_ can reach 83 mL/kg/min (Lucia et al., 2008). A study conducted in 2021 indicated that elite marathoners need to achieve a maximal oxygen uptake of 71.0 ± 5.7 mL/kg/min, an anaerobic threshold velocity of 18.9 ± 0.4 km/h, and an oxygen consumption of 191 ± 19 mL/kg/km to maintain a running speed of 21.1 km/h (Jones et al., 2021). However, data on anaerobic threshold velocities for men below 2:10:00 and for women below 2:25:00 are still lacking (Joyner et al., 2020).

Running economy plays a critical role in determining marathon speed while keeping energy expenditure in check, making it essential for elite marathon runners to possess an excellent running economy for success (Saunders et al., 2004). For elite runners with similar maximal oxygen uptake, running economy was a better predictor of marathon performance than maximal oxygen uptake. In the 1960s and 1970s, top marathoners Frank Shorter and Derek Clayton exhibited oxygen consumption ranging from 70-71 mL/kg/min, which aligns with the anaerobic threshold speed (Saltin et al., 1995). Limited data is available regarding running economy for top marathoners. Environmental and biomechanical factors also appear to affect the running economy of elite runners. Recent research has focused on enhancing the performance of elite marathoners by improving running economy through various means, such as utilizing shoes that are 100 g lighter, employing four lead runners to alternately block wind in the latter half of the race, or leveraging a tailwind of 6.0 m/s (Hoogkamer et al., 2017). Furthermore, studies on marathon shoes and the running economy have emerged as popular research topics, exploring improvements in landing time and suggesting that world-class athletes are the primary beneficiaries of enhanced footwear technology. In general, footwear technology has demonstrated moderate benefits in improving running economy (Muniz-Pardos et al., 2021).

### Elite marathon runners training intensity and pacing strategies

Elite distance runners typically undergo 8-10 years of prolonged exercise training before achieving international standards (Haugen et al., 2022). Prior research has shown that a moderate amount of high-intensity training (approximately 40 km/week) can stimulate the body’s potential for maximal exercise loads (Gibala et al., 2012). Additionally, several hours of daily exercise can enhance the anaerobic threshold velocity of elite marathon runners by increasing mitochondrial content and capillary density in their leg muscles (Hughes et al., 2018). Training at altitude and engaging in high-intensity training are effective methods for promoting mitochondrial catabolism and regeneration, leading to the production of more efficient mitochondria in the leg muscles of elite endurance athletes (King, 2021). A recent study involving top male and female marathoners who underwent 8 weeks of high-intensity training, covering distances of 180 ± 27 km and 155 ± 19 km, respectively, before a race revealed improved peakVO_2_ among the athletes. Although their running economy remained unchanged, they exhibited reduced peakVO_2_ utilization at a marathon pace (Doherty et al., 2020).

Pacing represents one of the primary factors influencing a marathoner’s performance on race day, and a consistent pace, determined by characteristic training volume, the number of races of the marathon distance, and average training pace, significantly contributes to enhanced marathon performance (Leslie et al., 2023). A perfectly uniform pace is highly desirable, with successful marathoners generally achieving a faster pace in the first half of the race and a slower pace in the second half. Comparing the pacing of marathon record holders from different eras, it was observed that those from 1988 to 2018 demonstrated relatively slower pacing in the first half and faster pacing in the second half, with less overall variation in pacing (Casado et al., 2021). This trend may continue in the future. Furthermore, an analysis of top-level runners during the 2016 Rio Olympics and the 2017 IAAF World Championships revealed that women exhibited less variation in pace changes compared to men throughout the race (Casado et al., 2021).

### Nutritional strategies for elite marathon runners

Nutrient supplementation is essential for athletes to achieve optimal athletic performance. Carbohydrates serve as the primary energy source for marathon runners, accounting for 90% of their energy expenditure, and their consumption has been linked to improved race performance (Stellingwerff, 2013). Elite marathon runners require rapid uptake of external glucose during running, insulin release to deliver glucose to muscle cells, electrolyte replenishment, and promotion of fluid retention (Ravindra et al., 2022). Incrementally increasing carbohydrate intake ensures the replenishment and maximization of muscle glycogen reserves. Typically, carbohydrate intake during marathon training ranges from 5 to 7 g/kg/day, with higher intake closer to the race (Drewnowski, 1998). While some studies have suggested that fat intake may help marathon runners conserve carbohydrates, it is unclear whether this contributes to endurance performance. Sufficient protein intake from external sources is necessary to maintain skeletal muscle mass, and Thomas and colleagues recommend that endurance athletes consume at least 20 g of protein every 3-4 h (Ravindra et al., 2022).

Marathon exercise is associated with significant water and electrolyte deficits, and dehydration-induced weight loss is a key factor in performance decline. Hydrating according to thirst is the best approach for maintaining proper hydration in athletes. Consuming 5-10 mL of carbohydrate-containing water per kilogram 2-4 h prior to exercise is also advised (Ravindra et al., 2022). Furthermore, hyponatremia, a serious complication resulting from faulty nutritional strategies, is associated with a BMI of less than 20 kg/m^2^, excessive water intake, and weight gain during competition (Schrier et al., 2013).

### Age and sex differences in marathon performance

The relationship between age and running time in elite marathoners follows a “U" shape, with faster running times observed between the ages of 15-25 and an increase in running times after the age of 35 (Lepers et al., 2020). Generally, peak endurance performance for marathon athletes occurs around the age of 35. As individuals age, cardiovascular and muscular function gradually declines, leading to a decrease in metabolism. This decline is associated with lower maximal heart rate, cardiac output, maximal oxygen uptake, running economy, and anaerobic threshold speed. Differences in maximal oxygen consumption contribute to 40%–77% of the variation in marathon performance, resulting in a decline of 7%-10% in endurance performance per decade for marathon runners (Trappe, 2007). However, studies suggest that elite or world-class master athletes can mitigate this decline through intense training. Some findings indicate that they can maintain a maximal oxygen uptake of 64.5 mL/kg/min at age 60 and 46.9 mL/kg/min at age 70, while also maintaining a comparable running economy to younger runners (Haugen et al., 2022). Additionally, high-level marathoners can sustain 90% of their maximal oxygen uptake for up to 2 h (Lepers et al., 2020).

With the increasing participation of women in marathons, gender differences in marathon performance have been studied since 1990 (Helgerud et al., 1990). Men generally exhibit faster running times due to physiological factors. Data from the Boston Marathon between 1972 and 2017 reveals that the annual 10 fastest men averaged an 18.3% faster time than women, with men also displaying higher maximal oxygen uptake compared to women (Knechtle et al., 2020). However, it is important to note that both men and women can maintain 90% of their maximal oxygen uptake during training when their performances are comparable (Helgerud et al., 1990). In a study comparing physiological indicators of 12 male and female runners with equivalent performances between the ages of 20 and 30, J. Helgerud found that performance-matched male and female marathoners exhibited similar maximal oxygen uptake (Helgerud et al., 1990). The difference lay in females having twice the amount of kilometers in their best 2-month training program but were less economical runners (Zavorsky et al., 2017). Recent studies indicate no significant difference in the age-related decline of physiological factors between male and female marathoners. However, marathon finishing times for female age group winners decline at a faster rate, approximately 27 s per year, after the age of 35 (Zavorsky et al., 2017).

### Recovery of inflammatory response and muscle damage

The heightened biomarkers of myocardial injury, such as cardiac troponin T (cTnT) and N-terminal pro-brain natriuretic peptide (NT-proBNP), subsequent to marathon exercise, have been a subject of considerable debate in the field. To date, no study has definitively clarified their clinical significance (Niemelä et al., 2016). A study conducted in 2006 involving 60 non-elite marathon runners, using pre- and post-event echocardiography and serum biomarkers, revealed that 40% of participants exhibited cardiac troponin levels equivalent to acute myocardial infarction (≥0.03 ng/mL) (Neilan et al., 2006). Subsequent studies indicated that elevated biomarkers, including cardiac troponin, normalized within 72 h (Scherr et al., 2011). This response was even more pronounced among amateur marathoners. Additionally, it is imperative to note sports-related sudden cardiac arrest (SCA). The Race Associated Cardiac Arrest Event Registry documented a higher risk of SCA among marathon runners compared to non-marathon participants, based on data from 10.9 million individuals involved in U.S. marathons between 1 January 2000, and 31 May 2010. Furthermore, males were found to have a greater incidence of SCA (0.90 vs 0.16/100,000) and accounted for 50% of all sudden cardiac deaths (SCD) in the last mile (Franklin et al., 2020).

Skeletal muscle damage commonly occurs as a complication of strenuous exercise, with studies revealing significant elevation of markers of skeletal muscle damage, such as lactate dehydrogenase (LDH), myoglobin, and creatine kinase (CK) following strenuous exercise (Bernat-Adell et al., 2021). Exercise-induced muscle damage (EIMD) can also impact running performance by altering stride length, consequently increasing the oxygen consumption rate while running. However, the potential alteration of other physiological parameters by EIMD remains uncertain. Furthermore, limited research has been conducted on the effect of exercise-induced muscle damage on marathon performance, particularly over longer durations and distances, indicating a crucial area for future investigation (Howatson and van Someren, 2008).

## Limitations

This study encountered several limitations. Firstly, it solely relied on literature sourced from the WoSCC database, excluding high-quality studies available in PubMed, Scopus, and other databases. Secondly, the study exclusively considered English literature, potentially introducing research bias by excluding studies in other languages.

## Conclusion

To the best of our knowledge, this is the first bibliometric study conducted in the field of marathon athletics. Our study aims to review the significant contributors, collaborative models, research hotspots, and trends observed in marathon performance publications over the past 15 years. Recent research focuses on marathon performance physiology, training strategies, nutrition, age and gender differences and inflammatory response. Overall, this study serves as a valuable reference for marathon performance researchers, providing insights into more focused future research directions, and informed scientific decisions.

## Abbreviations

CK, creatine kinase; EIMD, exercise-induced muscle damage; IAAF, the International Association of Athletics Federations; WOSCC, Web of Science Core Collection; peakVO2, maximum oxygen uptake; cTnT, cardiac troponin T; NT-proBNP, N-terminal pro-brain natriuretic peptide; SCA, sudden cardiac arrest; SCD, sudden cardiac deaths; LDH, lactate dehydrogenase.
