# Supplementary figures and images for: Themes and trends in marathon performance research: a comprehensive bibliometric analysis from 2009 to 2023
Source: Front Physiol. 2024 May 10;15:1388565. doi: 10.3389/fphys.2024.1388565 (PMC11116898; doi:10.3389/fphys.2024.1388565)

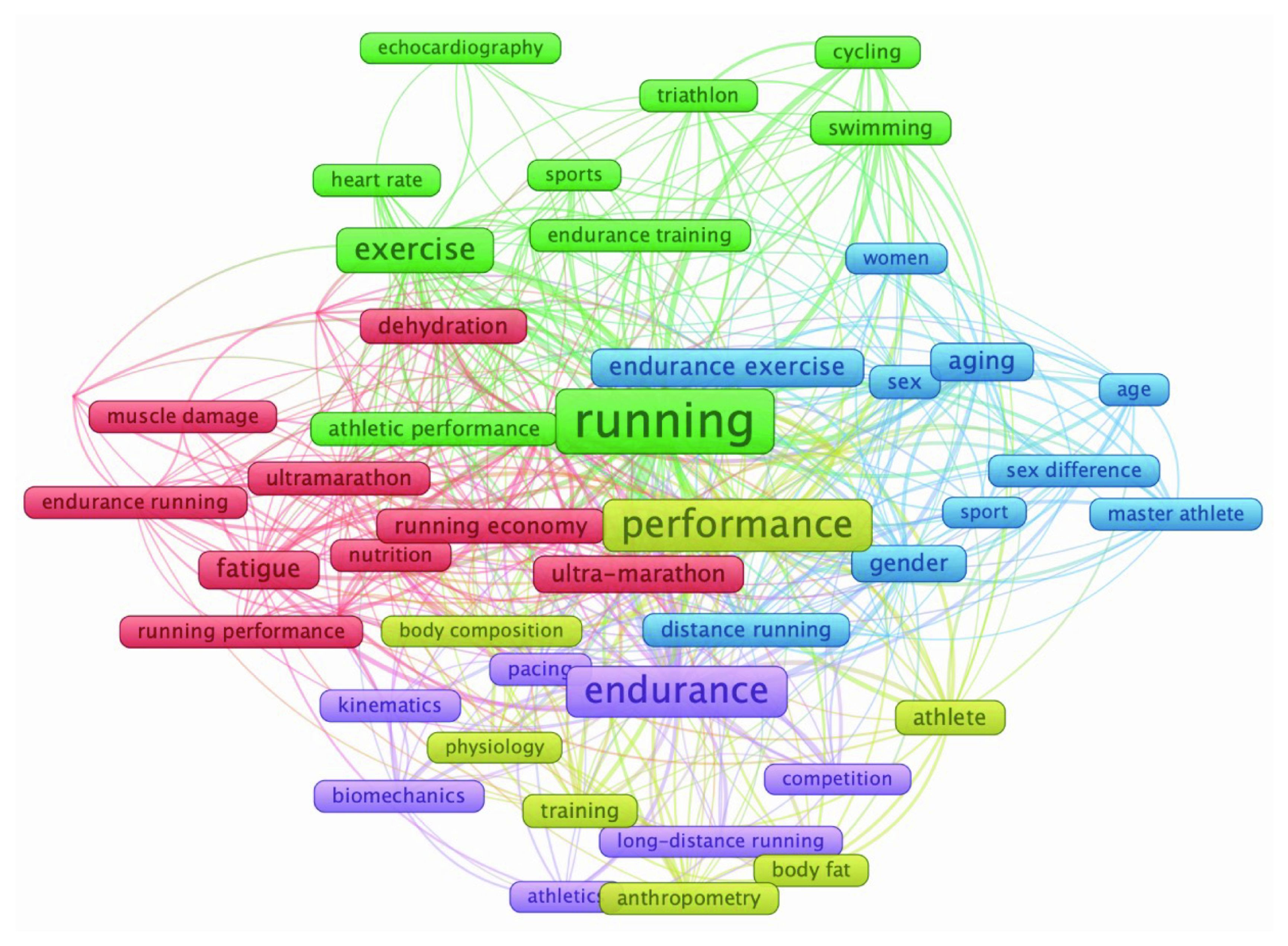

Supplement: Supplementary file 2 [file Image5.jpg]

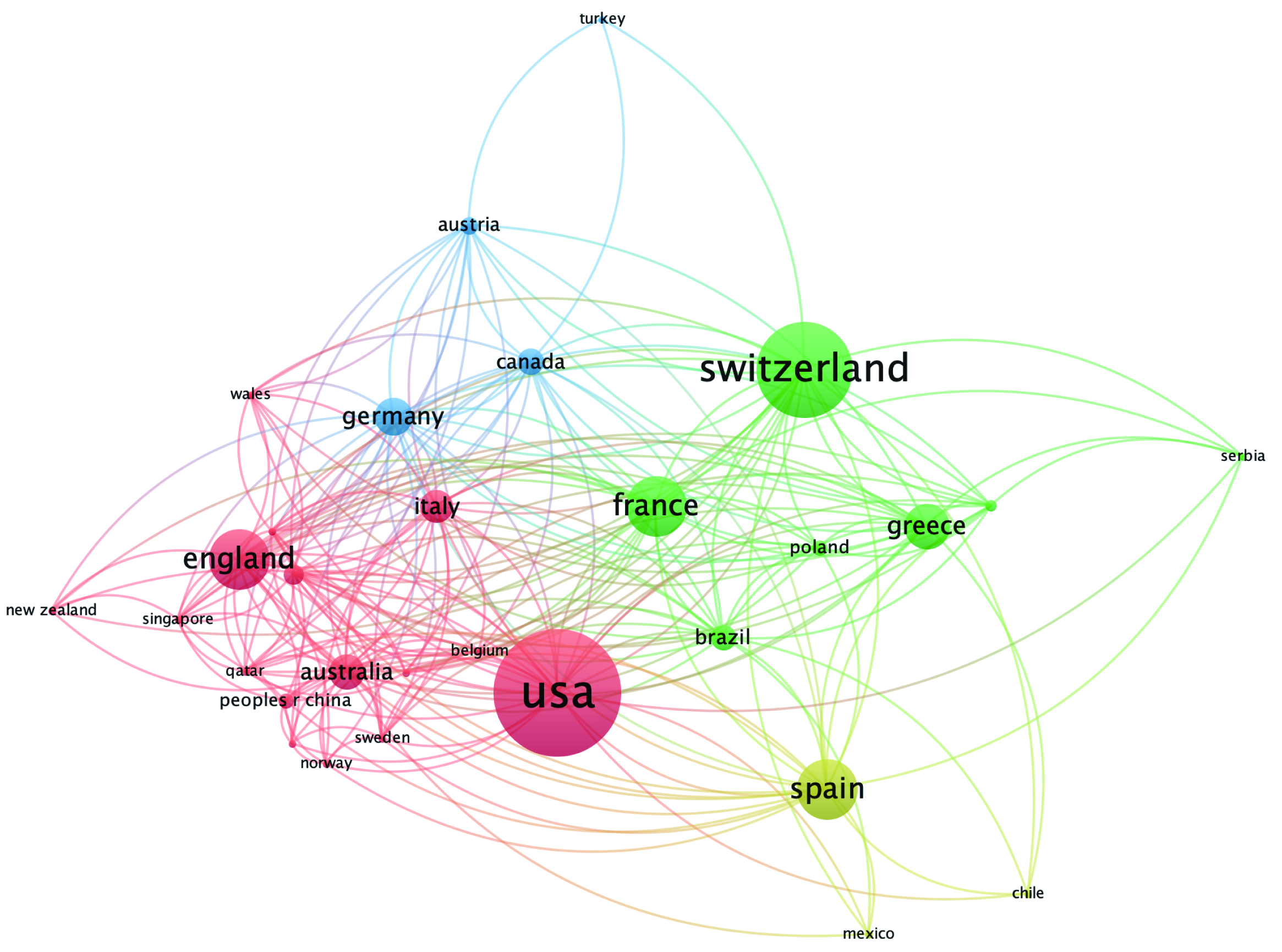

Supplement: Supplementary file 3 [file Image1.tiff]

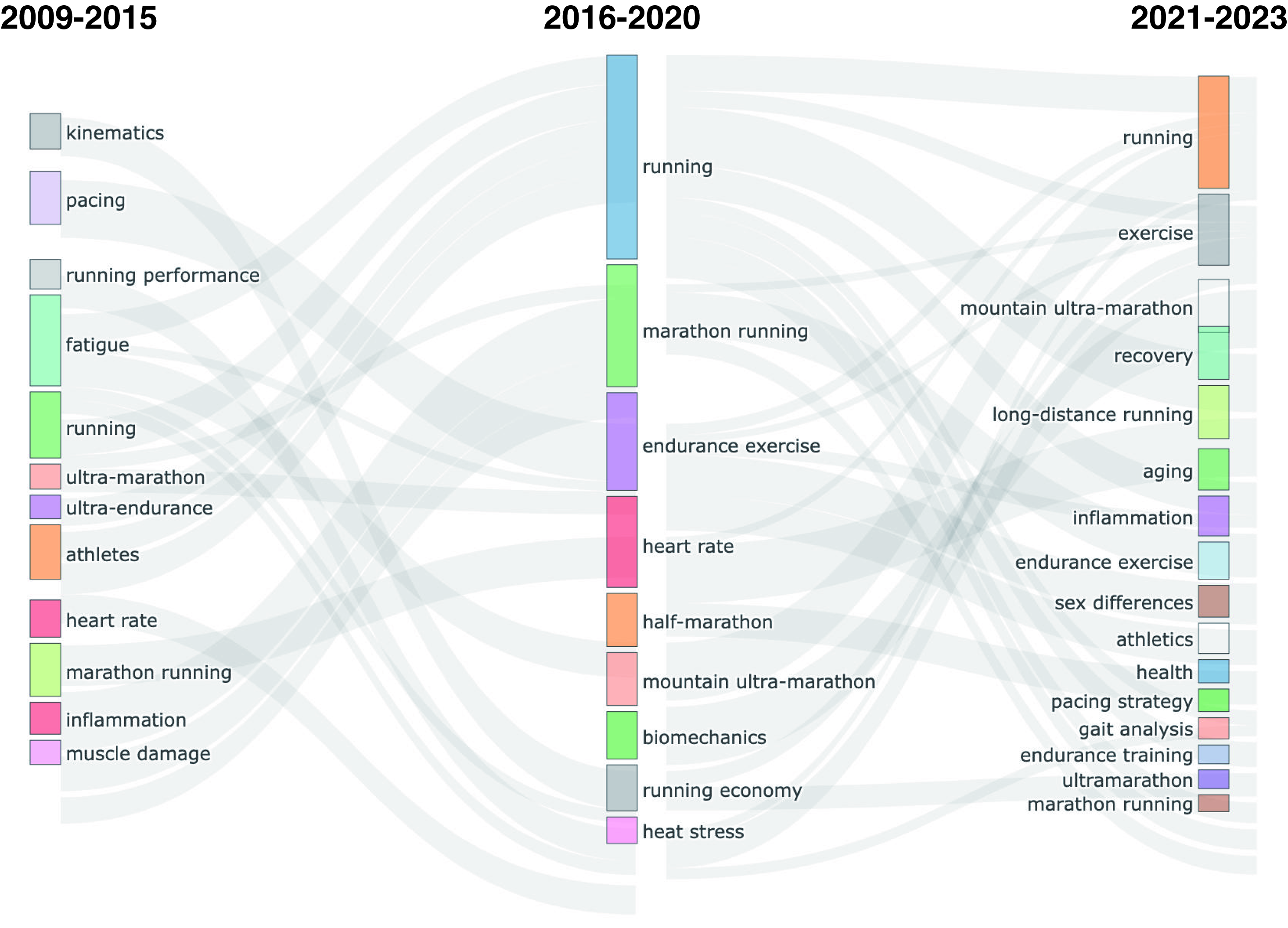

Supplement: Supplementary file 4 [file Image6.tif]

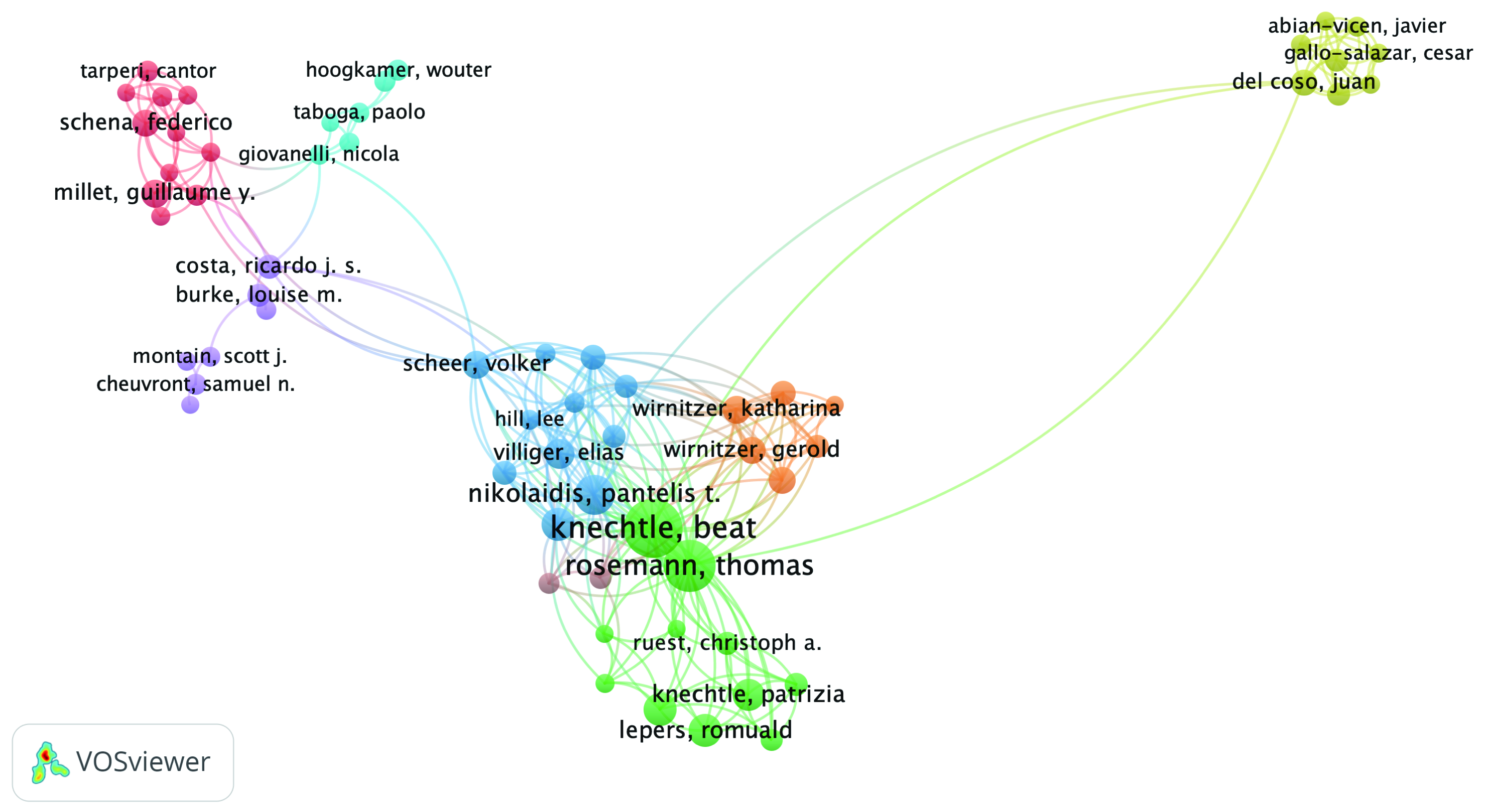

Supplement: Supplementary file 6 [file Image4.tif]

# Top 20 Keywords with the Strongest Citation Bursts

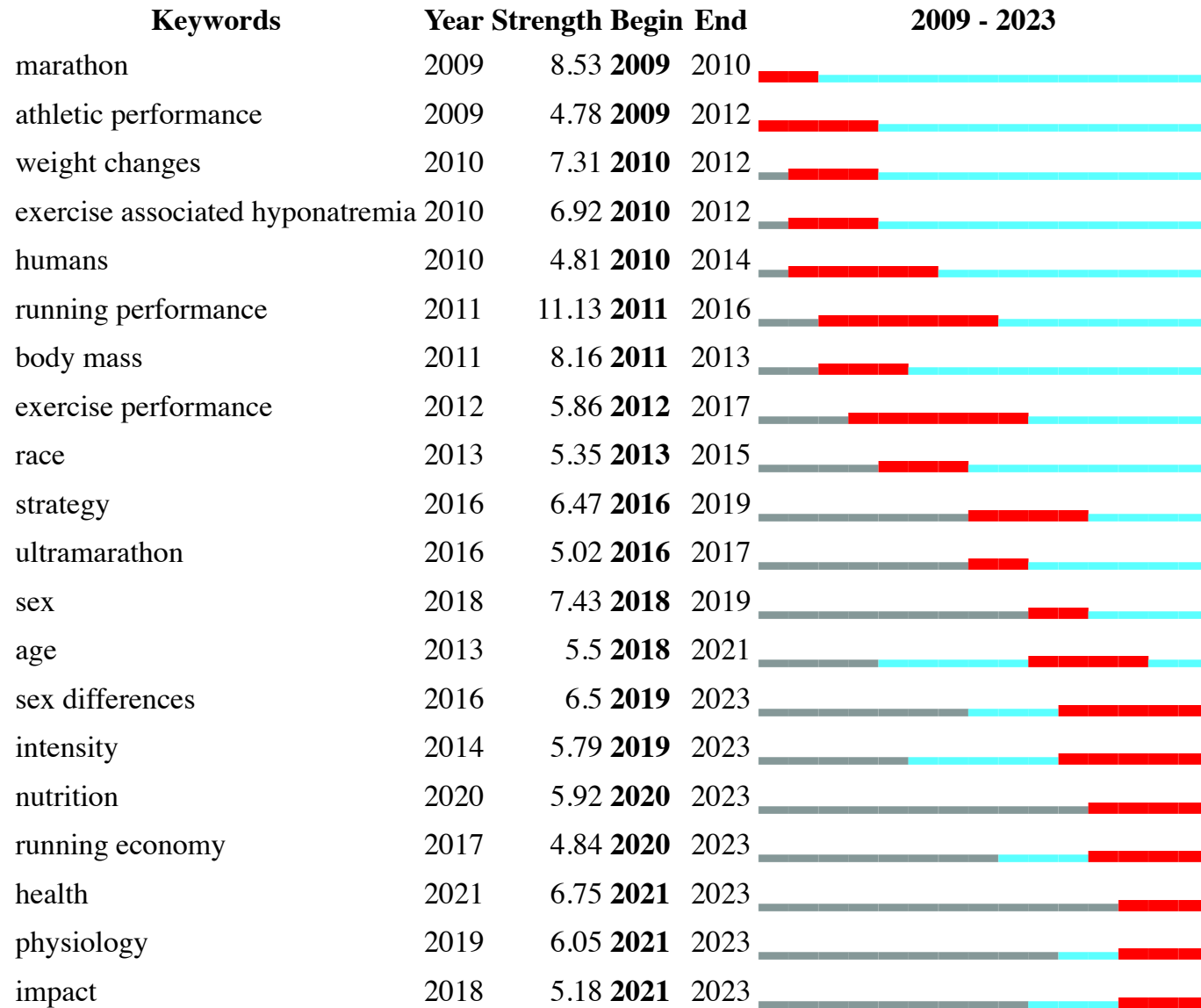

Supplement: Supplementary file 7 [file Image8.pdf]
