# Supplementary material for: Themes and trends in marathon performance research: a comprehensive bibliometric analysis from 2009 to 2023
Source: Front Physiol. 2024 May 10;15:1388565. doi: 10.3389/fphys.2024.1388565 (PMC11116898; doi:10.3389/fphys.2024.1388565)
Supplement: Supplementary file 5 [file Table1.docx]

**Supplementary Table 1.** The top 10 productive countries/regions

| Rank | Countries/regions | Publications | Citations |
| --- | --- | --- | --- |
| 1 | United States | 652 | 5133 |
| 2 | Switzerland | 423 | 3122 |
| 3 | Spain | 335 | 1208 |
| 4 | United Kingdom | 309 | 1780 |
| 5 | France | 275 | 1693 |
| 6 | Italy | 211 | 1102 |
| 7 | Germany | 207 | 1412 |
| 8 | China | 199 | 454 |
| 9 | Greece | 151 | 276 |
| 10 | Brazil | 135 | 276 |

**Supplementary Table 2.** The top 10 productive authors

| Rank | Authors | Publications | H-index | Citations |
| --- | --- | --- | --- | --- |
| 1 | Knechtle B | 171 | 29 | 2956 |
| 2 | Rosemann T | 117 | 26 | 2036 |
| 3 | Nikolaidis PT | 85 | 23 | 1261 |
| 4 | Rüst CA | 53 | 19 | 735 |
| 5 | Lepers R | 31 | 19 | 1060 |
| 6 | Knechtle P | 28 | 19 | 1186 |
| 7 | Villiger E | 25 | 13 | 710 |
| 8 | Schena F | 23 | 12 | 397 |
| 9 | Millet GY | 22 | 12 | 484 |
| 10 | Scheer V | 22 | 11 | 353 |

**Supplementary Table 3.** The top 10 most co-cited references

| **Rank** | **First author, year** | **Title** | **Citations (n)** | **Altmetric Attention Score(n)** |
| --- | --- | --- | --- | --- |
| 1 | Lepers R, 2012  [18] | Do older athletes reach limits in their performance during marathon running? | 67 | 1 |
| 2 | Ely MR, 2007  [21] | Impact of weather on marathon-running performance | 60 | 126 |
| 3 | Billat VL, 2001  [8] | Physical and training characteristics of top-class marathon runners | 59 | 6 |
| 4 | Abbiss CR, 2008  [22] | Describing and understanding pacing strategies during athletic competition | 58 | 13 |
| 5 | Sawka MN, 2007  [23] | American College of Sports Medicine position stand. Exercise and fluid replacement | 56 | 485 |
| 6 | Hunter SK, 2011  [24] | Is there a sex difference in the age of elite marathon runners? | 55 | 118 |
| 7 | Ely MR, 2008  [25] | Effect of ambient temperature on marathon pacing is dependent on runner ability | 52 | 3 |
| 8 | Hoffman MD, 2010  [26] | Historical analysis of participation in 161 km ultramarathons in North America | 52 | 1 |
| 9 | Jokl P, 2004  [27] | Master's performance in the New York City Marathon 1983-1999 | 49 | 0 |
| 10 | Cheuvront SN, 2005  [28] | Running Performance Differences between Men and Women | 48 | 13 |
