# Supplementary material for: Themes and trends in marathon performance research: a comprehensive bibliometric analysis from 2009 to 2023
Source: Front Physiol. 2024 May 10;15:1388565. doi: 10.3389/fphys.2024.1388565 (PMC11116898; doi:10.3389/fphys.2024.1388565)
Supplement: Supplementary file 8 [file Image7.pdf]

Top 15 References with the Strongest Citation Bursts

References	Year	Strength	Begin	End	2009 - 2023
Hoffman MD, 2010, INT J HIST SPORT, V27, P1877, DOI 10.1080/09523367.2010.494385, DOI	2010	8.55	**2011**	2015	
Hoffman MD, 2010, INT J SPORTS MED, V31, P31, DOI 10.1055/s-0029-1239561, DOI	2010	9.18	**2012**	2014	
Lepers R, 2012, AGE, V34, P773, DOI 10.1007/s11357-011-9271-z, DOI	2012	13.85	**2013**	2017	
Hunter SK, 2011, MED SCI SPORT EXER, V43, P656, DOI 10.1249/MSS.0b013e3181fb4e00, DOI	2011	11.36	**2013**	2016	
Hoffman MD, 2009, MED SCI SPORT EXER, V41, P2191, DOI 10.1249/MSS.0b013e3181a8d553, DOI	2009	8.72	**2013**	2014	
Cohen J, 2013, STATISTICAL POW ...... SCIENCES, VRevised edition, P0, DOI	2013	11.59	**2015**	2018	
Santos-Lozano A, 2014, INT J SPORTS MED, V35, P933, DOI 10.1055/s-0034-1367048, DOI	2014	8.2	**2016**	2019	
Lara B, 2014, AGE, V36, P1003, DOI 10.1007/s11357-013-9614-z, DOI	2014	8.3	**2017**	2018	
Nikolaidis PT, 2017, OPEN ACCESS J SPORTS, V8, P171, DOI 10.2147/OAJSM.S141649, DOI	2017	8.07	**2018**	2020	
Nikolaidis PT, 2018, RES SPORTS MED, V26, P86, DOI 10.1080/15438627.2017.1393752, DOI	2018	7.93	**2018**	2020	
Knechtle B, 2018, FRONT PHYSIOL, V9, P0, DOI 10.3389/fphys.2018.00634, DOI	2018	11.06	**2019**	2023	
Nikolaidis PT, 2018, CHINESE J PHYSIOL, V61, P85, DOI 10.4077/CJP.2018.BAG535, DOI	2018	8.15	**2019**	2021	
Scheer V, 2019, SPORTS MED ARTHROSC, V27, P3, DOI 10.1097/JSA.0000000000000198, DOI	2019	8.48	**2020**	2023	
Hoogkamer W, 2018, SPORTS MED, V48, P1009, DOI 10.1007/s40279-017-0811-2, DOI	2018	9.53	**2021**	2023	
Vitti A, 2020, RES SPORTS MED, V28, P121, DOI 10.1080/15438627.2019.1586705, DOI	2020	9.44	**2021**	2023	
